# Cycloastragenol improves tolerance to acute hypoxic exhaustive exercise stress in mice: integrated network pharmacology and experimental evidence

**DOI:** 10.3389/fnut.2026.1856961

**Published:** 2026-07-23

**Authors:** Dan Liu, Yiming Zhang, Minghui Jia, Yimeng Gu, Xuefeng Xi, Yi Zhang

**Affiliations:** 1 Southwest Jiaotong University, Chengdu, China; 2 Henan University, Kaifeng, China

**Keywords:** acute hypoxia, *Astragalus membranaceus*, cycloastragenol, exhaustive exercise, oxidative stress

## Abstract

**Background:**

Acute hypoxic exposure combined with exhaustive exercise can impair exercise performance and induce metabolic, inflammatory, oxidative, and tissue stress. Cycloastragenol, a bioactive constituent of *Astragalus membranaceus*, has shown antioxidant and tissue-protective potential, but its role in acute hypoxic exercise stress remains unclear. This study investigated whether cycloastragenol pretreatment attenuates fatigue- and injury-related responses in mice.

**Methods:**

A combined exploratory network pharmacology and animal experimental approach was used. Eighty-four male C57BL/6 J mice were randomly assigned to seven groups: blank control, model control (MC), normoxic exercise, *Rhodiola rosea* positive control (300 mg/kg/day), and low-, medium-, and high-dose cycloastragenol groups (25, 50, and 100 mg/kg/day). Pretreatments were administered by oral gavage once daily for 40 days. The MC, positive-control, and cycloastragenol groups then underwent continuous 24-h simulated hypobaric hypoxia equivalent to 5,000 m, followed by treadmill running to exhaustion. Outcomes included exhaustion time, serum biochemical and inflammatory markers, gastrocnemius ATPase activities, representative histopathology, and selected protein-expression endpoints. Data were analyzed using one-way ANOVA with Dunnett’s test, eta squared, and Cohen’s *d*.

**Results:**

Network pharmacology identified 116 overlapping targets and suggested HIF-1, PI3K-Akt, FoxO, and IL-17 signaling as hypothesis-generating pathways. Compared with MC, high-dose cycloastragenol prolonged exhaustion time (133.67 ± 7.45 vs. 96.92 ± 9.42 min; *d* = +4.33; *p* < 0.001), reduced BUN (16.14 ± 1.21 vs. 23.38 ± 1.61 mmol/L; *d* = −5.09; *p* < 0.001), lactate (7.72 ± 1.77 vs. 16.27 ± 2.62 mmol/L; *d* = −3.82; *p* < 0.001), MDA (1.08 ± 0.07 vs. 1.20 ± 0.10 nmol/mg; *d* = −1.41; *p* < 0.01), IL-6 (14.92 ± 1.00 vs. 28.69 ± 1.41 pg./mL; *p* < 0.001), and TNF-α (51.31 ± 2.18 vs. 114.93 ± 1.84 ng/mL; *p* < 0.001), while increasing T-SOD activity and Na + -K + -ATPase activity. Representative HE images and Western blot results suggested qualitative tissue protection and modulation of HIF-1alpha, FoxO1, Nrf2, and HO-1 expression.

**Conclusion:**

Cycloastragenol pretreatment was associated with improved tolerance to acute hypoxic exhaustive exercise and attenuation of metabolic, inflammatory, oxidative, and tissue-related responses in mice. Direct hepatic glycogen, muscle glycogen, and blood glucose endpoints were not measured; further mechanistic and translational studies are needed.

## 1 Introduction

Acute hypoxic exposure is a substantial physiological challenge that can impair oxygen transport and reduce exercise capacity (1). When hypoxia is combined with exhaustive exercise, this challenge may be further intensified, leading to metabolic disturbance, oxidative stress, inflammatory activation, and structural injury in multiple tissues, particularly skeletal and cardiac muscle (2). These responses are closely linked to reduced exercise tolerance and fatigue development under hypoxic conditions (3). Therefore, identifying safe and effective nutritional or bioactive interventions that improve tolerance to hypoxic exercise stress is of both physiological and practical relevance.

Natural bioactive compounds have attracted increasing attention as potential nutritional strategies for stress adaptation and tissue protection (4). Cycloastragenol, an active constituent derived from *Astragalus membranaceus*, has been reported to possess anti-inflammatory, antioxidative, and tissue-protective properties (5). Existing evidence suggests that cycloastragenol may contribute to the regulation of redox homeostasis, attenuation of inflammatory responses, and protection against stress-related tissue injury (6). However, its potential role as a bioactive nutritional candidate in hypoxia-related exhaustive exercise stress remains insufficiently understood.

*Astragalus membranaceus* has long been used as a medicinal and food-related botanical material in traditional Chinese medicine (7), where it is commonly associated with “Qi-tonifying,” stress adaptation, and fatigue-related conditions. Cycloastragenol is a tetracyclic triterpenoid sapogenin and an aglycone/metabolite related to astragaloside IV, one of the major saponins of *Astragalus membranaceus* (8). Compared with whole Astragalus extracts (9), cycloastragenol provides a chemically more defined intervention, which is advantageous for mechanistic interpretation and reproducibility. Therefore, cycloastragenol was selected in the present study as a representative Astragalus-derived bioactive constituent for evaluating protection against acute hypoxic exercise stress.

The protective effects of cycloastragenol under hypoxic exercise stress are likely to involve multiple biological processes rather than a single pathway. Mechanistically, pathways related to hypoxic adaptation, oxidative stress regulation, inflammatory responses, and tissue injury may all be relevant (10–12). In particular, hypoxia-inducible factor-1 (HIF-1), phosphoinositide 3-kinase/protein kinase B (PI3K-Akt), forkhead box O (FoxO), and interleukin-17 (IL-17) signaling may contribute to the physiological response to hypoxic exhaustive exercise and may represent plausible pathways through which cycloastragenol exerts its bioactive effects.

Therefore, the present study aimed to investigate whether cycloastragenol pretreatment could attenuate fatigue and tissue injury induced by acute hypoxic exhaustive exercise in mice. To address this question, we combined mechanistic prediction with experimental validation and evaluated exercise performance, biochemical and inflammatory markers, histopathological alterations, and protein expression in brain, skeletal muscle, and myocardial tissues. We hypothesized that cycloastragenol pretreatment would improve tolerance to hypoxia-related exhaustive exercise stress, potentially through coordinated regulation of hypoxia-, inflammation-, and oxidative stress-related pathways.

## 2 Materials and methods

### 2.1 Reagents and chemicals

Cycloastragenol was used as the primary bioactive intervention compound (Jiangsu Yongjian Pharmaceutical Technology Co., Ltd., China; batch No. 231001; catalog No. 104392-231001; purity > = 98.0%), and *Rhodiola rosea* capsules were used as the positive control (Qinghai Weikang Plateau Health, China; batch No. 230703; catalog No. 01409; 0.3 g/capsule, containing 1.0 g salidroside per 100 g) (13). The administration vehicle was 0.5% carboxymethyl cellulose sodium (CMC-Na) prepared in normal saline. CMC-Na was purchased from Tianjin Kaitong Chemical Reagent Co., Ltd. Cycloastragenol and *Rhodiola rosea* preparations were freshly prepared in the vehicle before administration. L-lactate (LAC; A019-2-2), blood urea nitrogen (BUN; C013-2-1), total superoxide dismutase (T-SOD; A001-1-2), malondialdehyde (MDA; A003-1-2), and ATPase (A070-6-2) assay kits were obtained from Nanjing Jiancheng Bioengineering Institute (Nanjing, China). Mouse tumor necrosis factor-alpha (TNF-α) enzyme-linked immunosorbent assay (ELISA) kits (E-EL-M3063) were obtained from Elabscience. Tissue fixative (G1111), hematoxylin and eosin (HE) staining solution (G1005), HIF-1α antibody (GB111339), Nrf2 antibody (GB113808), HRP-conjugated goat anti-rabbit IgG (GB23303), and HRP-conjugated goat anti-mouse IgG (GB23301) were obtained from Servicebio (Wuhan, China). HO-1 antibody (10701-1-AP) and FoxO1 antibody (66457-1-Ig) were obtained from Proteintech.

### 2.2 Network pharmacology analysis

A network pharmacology approach was used to explore potential targets and pathways through which cycloastragenol may influence fatigue- and hypoxia-related stress responses (14). Initial database searches were performed on August 31, 2024, and Supplementary material (exports/checks) were prepared from the available source records in May 2025 and organized for the present revision. Cycloastragenol-related targets were retrieved from SwissTargetPrediction, PharmMapper, and Super-PRED using the PubChem/SMILES information for cycloastragenol. Disease- and model-related targets were retrieved from GeneCards, OMIM, TTD, and PharmGKB using fatigue-related and hypoxia/model-related terms. Gene symbols were standardized before duplicate removal, and overlapping targets were identified as candidate targets for subsequent analysis. The original target-prediction files, disease/model target tables, overlap files, and database access notes are provided as Supplementary material.

The overlapping targets were imported into the STRING database using *Homo sapiens* gene identifiers to construct a protein–protein interaction network, which was further analyzed and visualized using Cytoscape. Core targets were screened based on topological parameters, including degree centrality, betweenness centrality, closeness centrality, network centrality, eigenvector centrality, and local average connectivity. Functional enrichment analyses, including gene ontology (GO) and Kyoto Encyclopedia of Genes and Genomes (KEGG) pathway analyses, were performed using DAVID output tables that include nominal *p-*values and multiple-testing correction columns, including Bonferroni, Benjamini, and FDR. Because the network analysis was based on human gene symbols and *Homo sapiens* STRING identifiers, and because not all predicted pathways were experimentally tested in mice, these findings were interpreted as hypothesis-generating rather than confirmatory.

### 2.3 Animals and experimental grouping

A total of 84 male C57BL/6 J mice aged 8 weeks were used in this study. After acclimatization, all male C57BL/6 J mice were numbered and allocated to seven groups using a random-number table: blank control group (BC, *n* = 12), model control group (MC, *n* = 12), normoxic exercise group (EX, *n* = 12), positive control group (PC, *n* = 12), low-dose cycloastragenol group (LD, *n* = 12), medium-dose cycloastragenol group (MD, *n* = 12), and high-dose cycloastragenol group (HD, *n* = 12). The BC group served as the normal control, the MC group was used to establish the acute hypoxic exhaustive exercise model, the EX group served as a normoxic exercise reference, the PC group received positive control treatment, and the LD, MD, and HD groups received different doses of cycloastragenol pretreatment. Laboratory personnel who performed the biochemical assays, ELISA assays, ATPase assays, and Western blot quantification were blinded to group allocation by using coded samples. The treadmill exhaustion test was not fully blinded because group-specific handling was required during the experimental procedure. All animal procedures were approved by the Animal Ethics Committee of Henan University (approval No. HUSOM2024-252) and were conducted in accordance with the approved protocol.

### 2.4 Pretreatment and hypoxic exhaustive exercise protocol

Following randomization, the mice received the assigned pretreatments by oral gavage once daily at approximately 09:00 for 40 consecutive days. The BC, MC, and EX groups received 0.5% CMC-Na in normal saline. The PC group received *Rhodiola rosea* at 300 mg/kg/day. The LD, MD, and HD groups received cycloastragenol at 25, 50, and 100 mg/kg/day, respectively. Body weight was monitored every 3 days throughout the intervention period. The cycloastragenol dose range was selected based on previous *in vivo* studies and available safety information, with the aim of covering low, medium, and high pretreatment doses while avoiding overt toxicity (15). *Rhodiola rosea* was selected as a positive-control comparator because of its reported adaptogenic and exercise-performance-related properties (16, 17).

**Figure 1:**
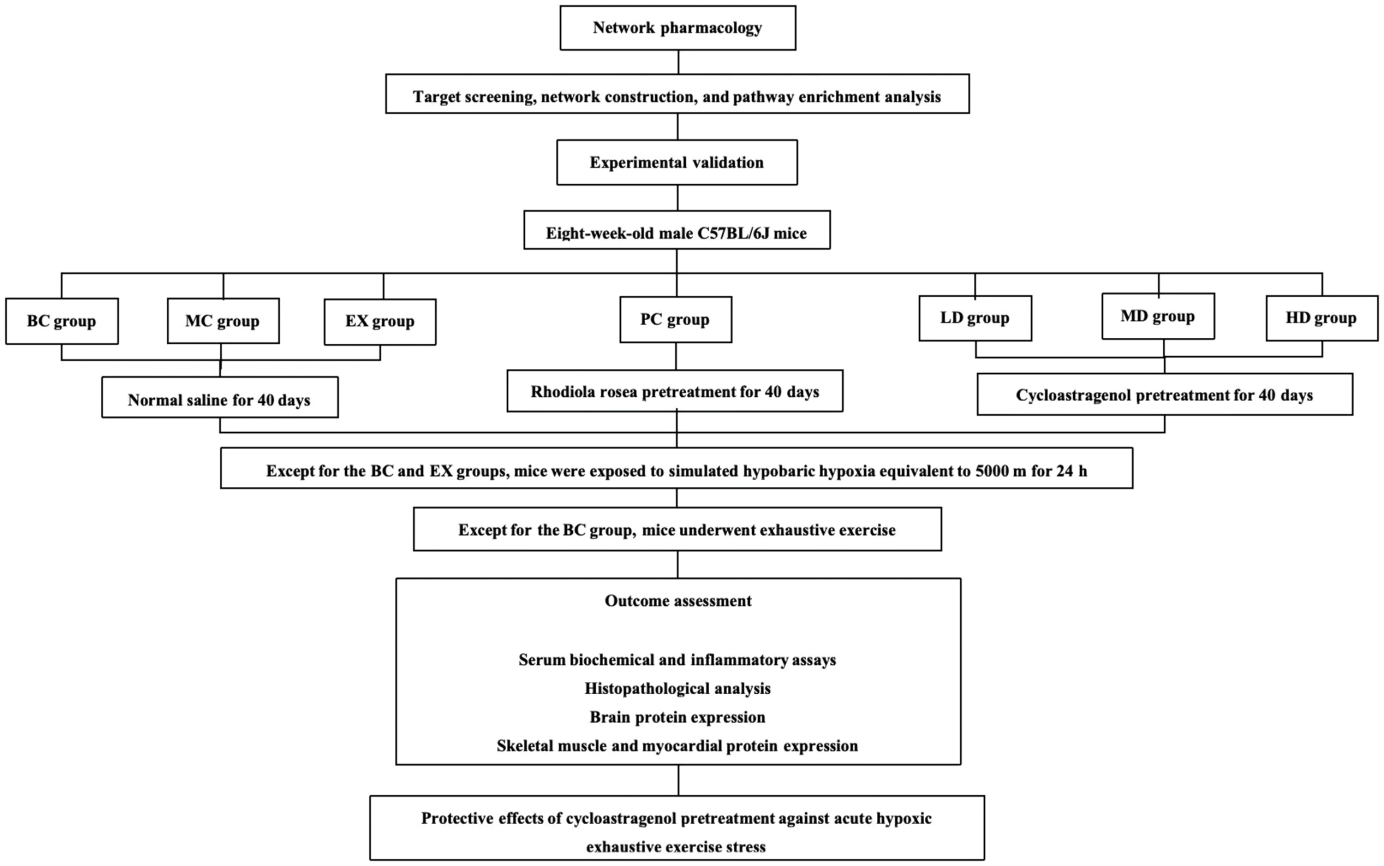
Study design and workflow.

After the pretreatment period, mice in the MC, PC, LD, MD, and HD groups were exposed to simulated hypobaric hypoxia equivalent to 5,000 m for a continuous 24 h in a hypoxia chamber (Pro0x-810 L-D, Shanghai Tawang, China), whereas the BC and EX groups were maintained under normoxic conditions. The chamber was operated with an oxygen partial pressure of approximately 11.1 ± 0.3 kPa, an ascent rate of 10 m/s, a temperature of 20 ± 2 °C, and a relative humidity of approximately 35% ± 5%. The chamber was not opened during the 24-h exposure, and food and water were available ad libitum. Mice were not fasted before hypoxia exposure, treadmill testing, or sample collection. The exhaustive exercise test was performed on an animal treadmill (FT-200, Chengdu Taimeng Technology Co., Ltd., China); no forced swimming test or tail weighting was used. The treadmill incline was set at 0 degrees throughout the test. Before the formal test, mice were familiarized with treadmill running for 1 week (10 min/day at 10 m/min). The formal treadmill protocol began at 10 m/min for 15 min, followed by 12 m/min for 15 min, 14 m/min for 15 min, and 20 m/min for 30 min; thereafter, speed was increased by 3 m/min every 10 min until exhaustion. Exhaustion was defined as remaining on the shock grid for more than 10 s without attempting to resume running. The same shock-grid setting and predefined exhaustion criterion were applied to all exercise groups; however, the exact current setting was not recorded in the original experimental log and is therefore acknowledged as a methodological limitation. After hypoxic exposure, all groups except the BC group underwent the exhaustive treadmill test, and time to exhaustion was recorded as an indicator of exercise tolerance under the corresponding experimental conditions. The overall study design is summarized in Figure 1.

### 2.5 Biochemical, inflammatory, and ATPase assays

Serum biochemical and inflammatory indices were evaluated immediately after the experimental intervention and exhaustive exercise challenge. At exhaustion, mice in the exercise groups were immediately weighed and blood was collected from the orbital sinus without a planned post-exercise recovery interval. Mice in the BC group were sampled at the corresponding time point. Before blood collection, mice were anesthetized with inhaled isoflurane. Whole blood was centrifuged at 3,000 g for 20 min, and the separated serum was stored at −80 degrees C until analysis. Serum LAC, BUN, T-SOD, and MDA were measured using commercial assay kits according to the manufacturers’ instructions (*n* = 12 per group). Serum interleukin-6 (IL-6) and TNF-α levels were determined by ELISA (18) (*n* = 3 per group).

To assess skeletal muscle ATPase-related metabolic function, gastrocnemius tissue was collected immediately after sacrifice for the measurement of Ca2 + -Mg2 + -ATPase and Na + -K + -ATPase activities (*n* = 6 per group). Mice were euthanized under deep anesthesia before tissue collection. These indices were analyzed using commercial assay kits according to the corresponding protocols. Together, these measurements were used to evaluate metabolic disturbance, oxidative stress, inflammatory responses, and ATPase-related muscle function under acute hypoxic exhaustive exercise conditions. Hepatic glycogen, muscle glycogen, and blood glucose were not measured in the present experiment; therefore, energy-substrate availability was not directly assessed. All biochemical, ELISA, and ATPase assays were performed according to the manufacturers’ protocols using coded samples. Standard curves, blank wells, and kit-provided or protocol-recommended controls were included where applicable.

Humane endpoints included the predefined treadmill exhaustion criterion, severe respiratory distress, persistent inability to move, loss of righting reflex, or lack of response to stimulation. No animal reached a humane endpoint other than the predefined exhaustion criterion during the experiment.

### 2.6 Histopathological and Western blot analyses

Gastrocnemius muscle and myocardial tissues were collected after the exhaustive exercise challenge for histopathological observation and protein expression analysis. For histopathological examination, tissue samples were fixed, routinely processed, embedded in paraffin, sectioned, and stained with hematoxylin and eosin. Representative images of gastrocnemius muscle and myocardium were used to compare morphological alterations among groups, with particular attention to tissue architecture, fiber arrangement, interstitial changes, and inflammatory cell infiltration. Because the available images could not be traced back to individual animals, the histological findings were presented as representative observations rather than formal animal-level semi-quantitative statistical results.

For Western blot analysis, brain, gastrocnemius muscle, and myocardial tissues were homogenized for protein extraction (*n* = 3 per group). HIF-1alpha expression was assessed in brain tissue. In gastrocnemius muscle and myocardium, the expression levels of HIF-1alpha, Nrf2, HO-1, and FoxO1 were evaluated. After electrophoretic separation and membrane transfer, the target proteins were incubated with the corresponding primary and secondary antibodies, and protein bands were visualized and quantified using standard immunoblotting procedures. Western blot quantification was performed using coded samples, and beta-actin, GAPDH, or beta-tubulin was used as the loading control for normalization according to the corresponding tissue panel. Full uncropped blot images and individual densitometry values are provided in the Supplementary material (Western blot data package). Because the Western blot endpoint had a limited effective sample size, these results were used to support association-level interpretation rather than causal pathway confirmation.

### 2.7 Statistical analysis

Statistical analyses were performed using GraphPad Prism 8. Continuous variables are presented as mean ± SD, and available individual raw values are provided in the Supplementary data files. Differences among groups were assessed using one-way ANOVA followed by Dunnett’s multiple-comparison test, with the MC group used as the main comparator for intervention effects. The BC versus MC comparison was used to confirm model establishment. *p* < 0.05 was considered statistically significant. Because no formal *a priori* power analysis was recorded for the original experiment, a sensitivity power analysis was added for the revised manuscript. For the primary behavioral endpoint, the design included seven groups and 84 mice in total (*n* = 12 per group). At alpha = 0.05 and 80% power, this design was able to detect a large omnibus one-way ANOVA effect of approximately Cohen’s *f* = 0.42. For an MC-versus-treatment comparison with *n* = 12 mice per group, 80% power corresponded to a standardized difference of approximately Cohen’s *d* = 1.20; the observed HD-versus-MC effect for exhaustion time was *d* = 4.33. Secondary endpoints with smaller effective sample sizes, including ATPase assays (*n* = 6 per group) and ELISA/Western blot analyses (*n* = 3 per group), were interpreted as supportive and exploratory. Overall ANOVA effect sizes were summarized as eta squared (SSbetween/SStotal). Pairwise effect sizes versus MC were summarized as Cohen’s d using the pooled SD; the sign of d indicates the direction of the treatment-group mean relative to MC. Because some endpoints had small effective sample sizes, variance estimation and formal assumption testing for these endpoints were limited, and the corresponding conclusions were interpreted cautiously.

Histopathological findings from hematoxylin and eosin staining were presented as representative observations and were not included in formal animal-level statistical analysis because the available images could not be matched to individual animals. All other quantitative results were analyzed based on the original experimental data.

## 3 Results

### 3.1 Network pharmacology prediction of cycloastragenol-related targets and pathways

Network pharmacology analysis identified 116 overlapping targets between cycloastragenol-related targets and model-relevant fatigue−/hypoxia-related stress-response targets. A protein–protein interaction network was subsequently constructed based on these overlapping targets, and topological analysis further screened 8 hub targets, including STAT3, NFKB1, CASP3, HSP90AB1, ESR1, HSP90AA1, MAPK1, and HIF1A (Table 1).

**Table 1:** Experimental grouping and treatment design.

Group	Description	Pretreatment (route, dose, vehicle, duration)	Hypoxia exposure	Exhaustive exercise
BC	Blank control	Oral gavage; 0.5% CMC-Na in normal saline; once daily for 40 days	No	No
MC	Model control	Oral gavage; 0.5% CMC-Na in normal saline; once daily for 40 days	Yes	Yes
EX	Normoxic exercise	Oral gavage; 0.5% CMC-Na in normal saline; once daily for 40 days	No	Yes
PC	Positive control	Oral gavage; *Rhodiola rosea* 300 mg/kg/day in 0.5% CMC-Na; 40 days	Yes	Yes
LD	Low-dose cycloastragenol	Oral gavage; cycloastragenol 25 mg/kg/day in 0.5% CMC-Na; 40 days	Yes	Yes
MD	Medium-dose cycloastragenol	Oral gavage; cycloastragenol 50 mg/kg/day in 0.5% CMC-Na; 40 days	Yes	Yes
HD	High-dose cycloastragenol	Oral gavage; cycloastragenol 100 mg/kg/day in 0.5% CMC-Na; 40 days	Yes	Yes

**Figure 2:**
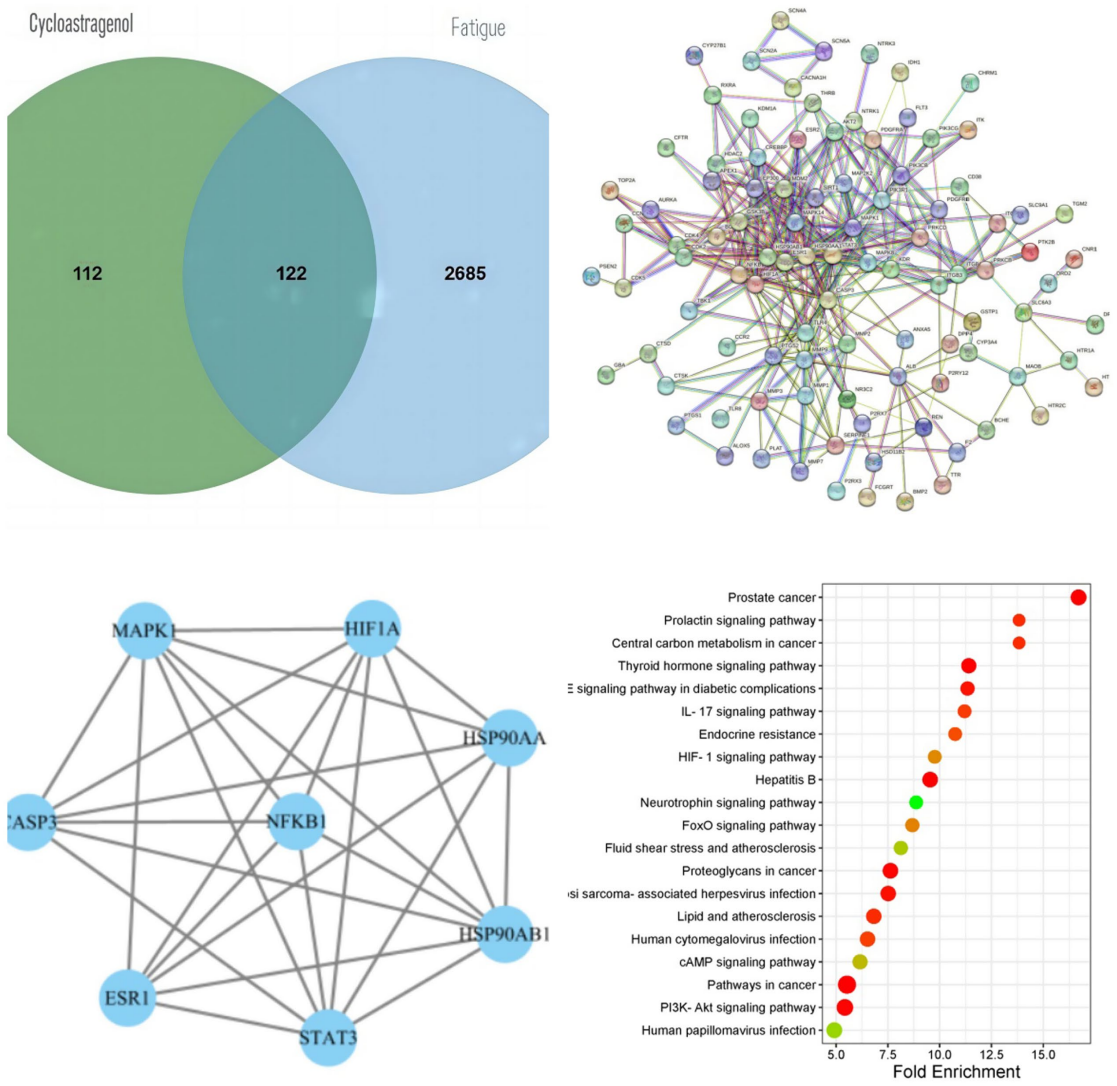
Network pharmacology analysis of cycloastragenol against model-relevant fatigue−/hypoxia-related stress-response targets.

GO enrichment analysis showed that these targets were associated with multiple biological processes, cellular components, and molecular functions related to stress responses. KEGG pathway enrichment analysis identified 147 pathways in total, of which 121 met the significance threshold of *p* < 0.01. Among the enriched pathways, HIF-1, PI3K-Akt, FoxO, and IL-17 signaling were highlighted as pathways of potential relevance to the present study (Figure 2).

Further examination of the pathway–target relationships showed that the HIF-1 signaling pathway involved STAT3, NFKB1, MAPK1, and HIF1A; the PI3K-Akt signaling pathway involved NFKB1, HSP90AB1, HSP90AA1, and MAPK1; the FoxO signaling pathway involved STAT3 and MAPK1; and the IL-17 signaling pathway involved HSP90AA1 and MAPK1. These pathway associations are summarized in Table 2.

**Table 2:** Core targets and key enriched pathways predicted by network pharmacology.

Pathway	Core targets involved	Biological implication
HIF-1 signaling pathway	STAT3, NFKB1, MAPK1, HIF1A	May be involved in hypoxic adaptation and the cellular response to reduced oxygen availability.
PI3K-Akt signaling pathway	NFKB1, HSP90AB1, HSP90AA1, MAPK1	May participate in cell survival, stress adaptation, and regulation of injury-related responses.
FoxO signaling pathway	STAT3, MAPK1	May be associated with oxidative stress regulation, metabolic adaptation, and tissue injury responses.
IL-17 signaling pathway	HSP90AA1, MAPK1	May be related to inflammatory activation and stress-induced tissue damage.

### 3.2 Effects of cycloastragenol pretreatment on exhaustive exercise performance

Following exposure to simulated hypobaric hypoxia and the exhaustive exercise challenge, exhaustion time showed a large group effect (*F*(5, 66) = 56.04; eta squared = 0.809). Compared with the MC group (96.92 ± 9.42 min), cycloastragenol pretreatment prolonged exhaustion time in the LD (107.92 ± 8.46 min; +11.3%; *d* = +1.23; *p* < 0.05), MD (111.67 ± 4.74 min; +15.2%; *d* = +1.98; *p* < 0.05), and HD groups (133.67 ± 7.45 min; +37.9%; *d* = +4.33; *p* < 0.001). The PC group also exhibited a longer exhaustion time than the MC group (126.00 ± 11.81 min; +30.0%; *d* = +2.72; *p* < 0.001), whereas the EX group showed the longest exercise time under normoxic conditions (155.25 ± 13.62 min; +60.2%; *d* = +4.98; *p* < 0.001). No obvious adverse changes in body weight were observed during the intervention period (overall eta squared = 0.036; Appendix 1).

### 3.3 Effects of cycloastragenol on metabolic disturbance and oxidative stress

Compared with the BC group, the MC group showed higher serum BUN (23.38 ± 1.61 vs. 8.01 ± 0.88 mmol/L), lactate (16.27 ± 2.62 vs. 9.10 ± 1.39 mmol/L), and MDA (1.20 ± 0.10 vs. 1.00 ± 0.07 nmol/mg), together with lower T-SOD activity (87.33 ± 10.71 vs. 120.79 ± 5.77 U/mL), indicating marked metabolic disturbance and oxidative stress after acute hypoxic exhaustive exercise. Overall group effects were large for BUN (eta squared = 0.944), lactate (eta squared = 0.681), T-SOD (eta squared = 0.547), and MDA (eta squared = 0.499).

Compared with MC, cycloastragenol pretreatment reduced BUN in the LD (20.83 ± 2.34 mmol/L; −10.9%; *d* = −1.27; *p* < 0.01), MD (19.45 ± 0.75 mmol/L; −16.8%; *d* = −3.14; *p* < 0.001), and HD groups (16.14 ± 1.21 mmol/L; −31.0%; *d* = −5.09; *p* < 0.001). Lactate was also lower in the cycloastragenol groups, most clearly in MD (8.41 ± 1.37 mmol/L; −48.3%; *d* = −3.76; *p* < 0.001) and HD (7.72 ± 1.77 mmol/L; −52.5%; *d* = −3.82; *p* < 0.001). For antioxidant and lipid-peroxidation markers, HD increased T-SOD activity (125.34 ± 10.60 U/mL; +43.5%; *d* = +3.57; *p* < 0.001) and reduced MDA (1.08 ± 0.07 nmol/mg; −10.5%; *d* = −1.41; *p* < 0.01) compared with MC. The PC group showed a similar direction of change for these markers. These results indicate that cycloastragenol pretreatment, particularly at the high dose, alleviated acute hypoxic exhaustive exercise-induced metabolic disturbance and oxidative stress in mice.

### 3.4 Effects of cycloastragenol on inflammatory responses

Compared with the BC group, the MC group showed markedly increased serum IL-6 (28.69 ± 1.41 vs. 10.50 ± 0.83 pg./mL) and TNF-α (114.93 ± 1.84 vs. 38.93 ± 2.66 ng/mL), indicating a pronounced inflammatory response after acute hypoxic exhaustive exercise. Overall group effects were large for both IL-6 (eta squared = 0.976) and TNF-α (eta squared = 0.989).

**Figure 3:**
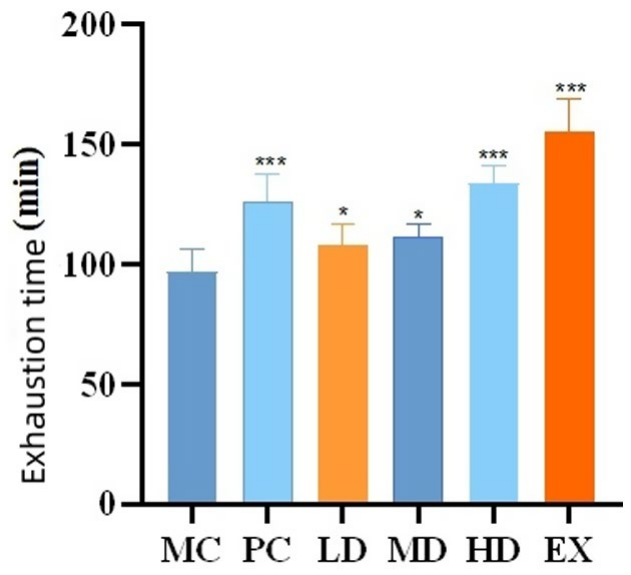
Effects of cycloastragenol pretreatment on exhaustive exercise performance. Exhaustion time in the MC, PC, LD, MD, HD, and EX groups following the experimental intervention and exhaustive exercise challenge (*n* = 12 per group). Data are presented as mean ± SD. Statistical significance is indicated in the figure. MC, model control; PC, positive control; LD, low-dose cycloastragenol; MD, medium-dose cycloastragenol; HD, high-dose cycloastragenol; EX, normoxic exercise.

Compared with MC, cycloastragenol pretreatment reduced IL-6 most clearly in the MD (22.39 ± 1.60 pg./mL; −22.0%; *p* < 0.001) and HD groups (14.92 ± 1.00 pg./mL; −48.0%; *p* < 0.001). TNF-α was reduced in the LD (99.38 ± 4.75 ng/mL; −13.5%; *p* < 0.01), MD (70.80 ± 4.06 ng/mL; −38.4%; *p* < 0.001), and HD groups (51.31 ± 2.18 ng/mL; −55.4%; *p* < 0.001). Because these ELISA endpoints had small effective sample sizes (*n* = 3 per group), pairwise Cohen’s d estimates were considered unstable and were therefore not emphasized in the main text; overall ANOVA effect sizes are reported above using eta squared. These results indicate that cycloastragenol pretreatment attenuated the inflammatory response induced by acute hypoxic exhaustive exercise in mice (see Figure 3).

### 3.5 Effects of cycloastragenol on gastrocnemius ATPase activities

**Figure 4:**
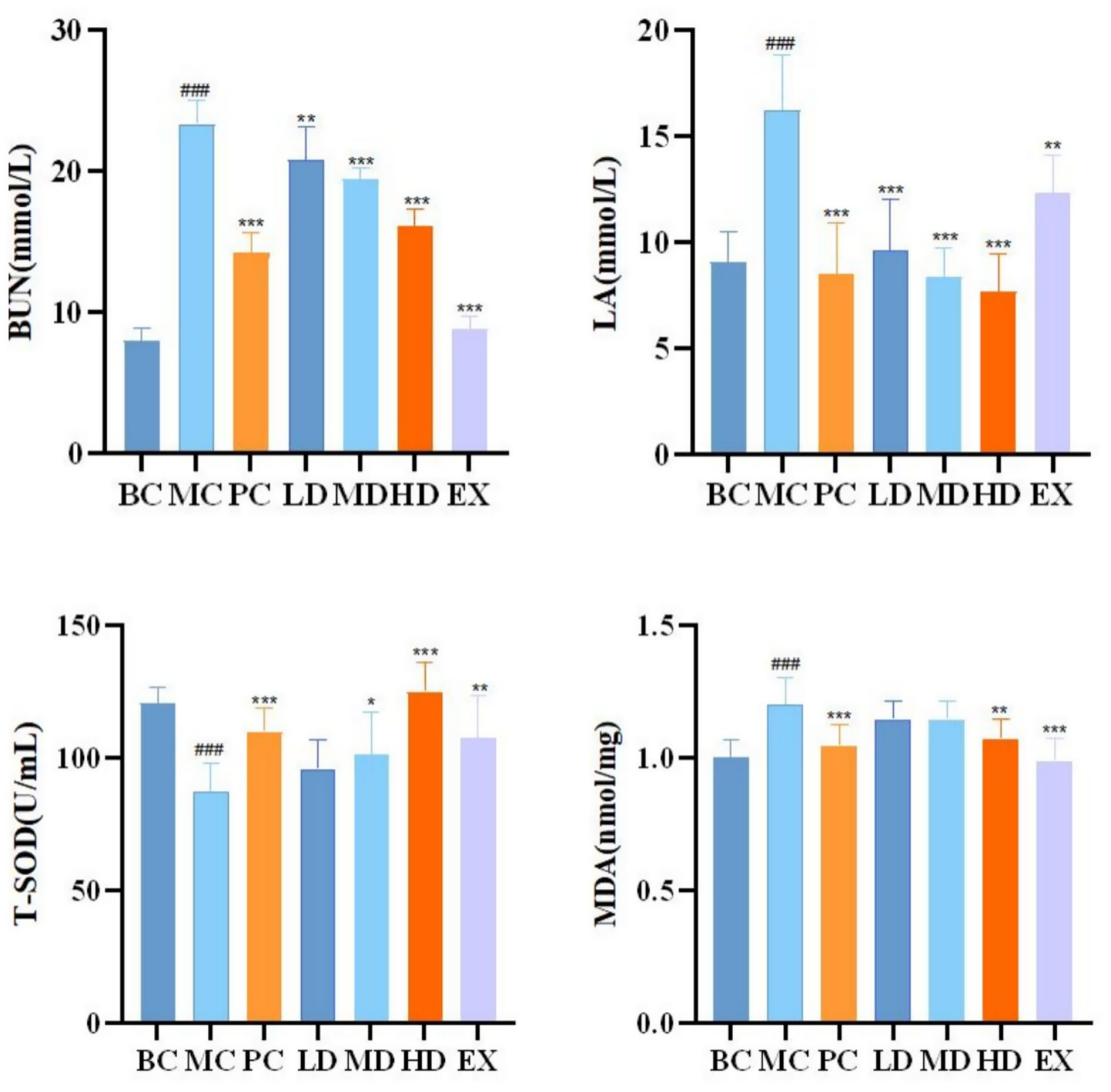
Effects of cycloastragenol on metabolic and oxidative stress-related indicators. **(A)** BUN, **(B)** LAC, **(C)** T-SOD, **(D)** MDA. Data are presented as mean ± SD (*n* = 12 per group). ## and ### indicate *p* < 0.01 and *p* < 0.001 versus the BC group, respectively; *, **, and *** indicate *p* < 0.05, *p* < 0.01, and *p* < 0.001 versus the MC group, respectively. BC, blank control; MC, model control; PC, positive control; LD, low-dose cycloastragenol; MD, medium-dose cycloastragenol; HD, high-dose cycloastragenol; EX, normoxic exercise.

**Figure 5:**
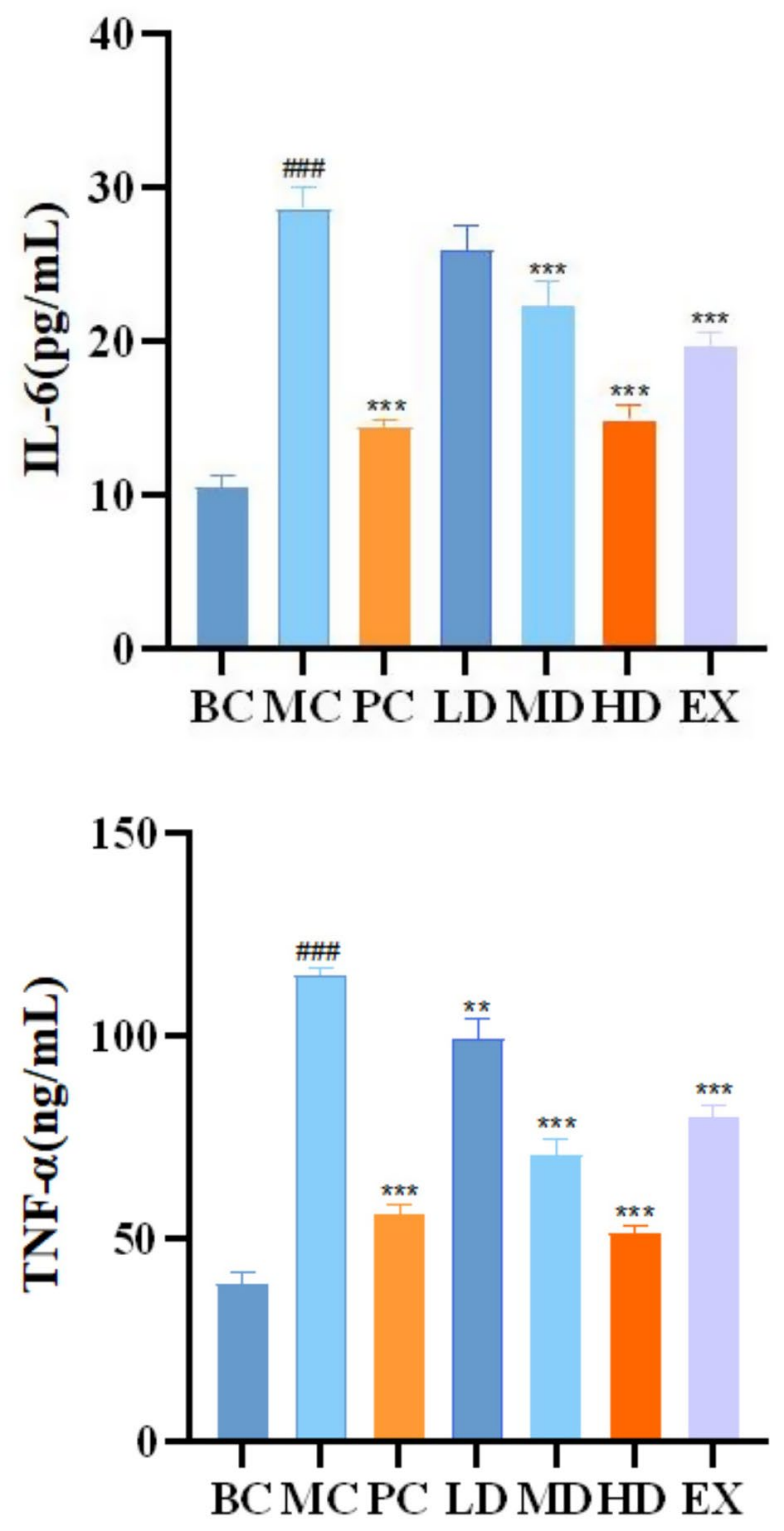
Effects of cycloastragenol on inflammatory responses. **(A)** IL-6. **(B)** TNF-α. Data are presented as mean ± SD (*n* = 3 per group). ### indicates *p* < 0.001 versus the BC group, and ** and *** indicate *p* < 0.01 and *p* < 0.001 versus the MC group, respectively. BC, blank control; MC, model control; PC, positive control; LD, low-dose cycloastragenol; MD, medium-dose cycloastragenol; HD, high-dose cycloastragenol; EX, normoxic exercise.

For gastrocnemius Ca2 + -Mg2 + -ATPase activity, the MC group was numerically higher than BC rather than lower (10.27 ± 1.17 vs. 8.78 ± 1.64 U/mgprot). Compared with MC, Ca2 + -Mg2 + -ATPase activity was further increased in the PC (12.22 ± 1.02 U/mgprot; +19.0%; *d* = +1.77; *p* < 0.05) and HD groups (12.87 ± 1.15 U/mgprot; +25.3%; *d* = +2.24; *p* < 0.01), with a large overall group effect (eta squared = 0.545) (see Figures 4, 5).

**Figure 6:**
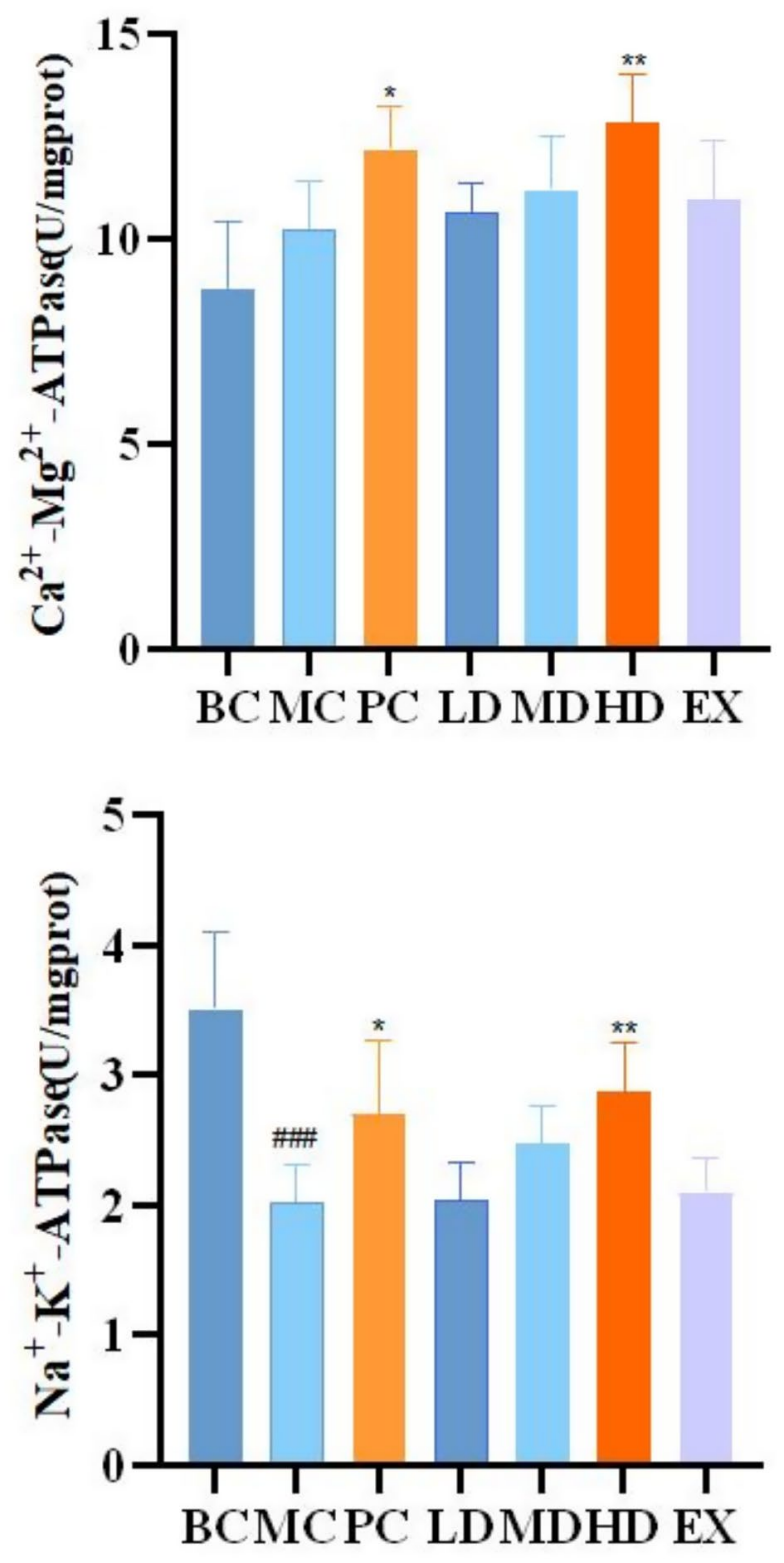
Effects of cycloastragenol on gastrocnemius ATPase activities. **(A)** Ca2 + -Mg2 + -ATPase. **(B)** Na + -K + -ATPase. Data are presented as mean ± SD (*n* = 6 per group). ## and ### indicate *p* < 0.01 and *p* < 0.001 versus the BC group, respectively; * and ** indicate *p* < 0.05 and *p* < 0.01 versus the MC group, respectively. BC, blank control; MC, model control; PC, positive control; LD, low-dose cycloastragenol; MD, medium-dose cycloastragenol; HD, high-dose cycloastragenol; EX, normoxic exercise.

For Na + -K + -ATPase activity, the MC group was lower than BC (2.03 ± 0.29 vs. 3.51 ± 0.58 U/mgprot), consistent with impaired ion-transport-related muscle function after acute hypoxic exhaustive exercise. Compared with MC, HD cycloastragenol increased Na + -K + -ATPase activity to 2.88 ± 0.37 U/mgprot (+41.8%; *d* = +2.55; *p* < 0.01), and the PC group showed a similar increase (2.71 ± 0.56 U/mgprot; +33.7%; *d* = +1.53; *p* < 0.05). These findings are shown in Figure 6 and indicate that the ATPase-related response was most consistent for Na + -K + -ATPase and high-dose cycloastragenol.

### 3.6 Histopathological changes in gastrocnemius muscle and myocardium

**Figure 7:**
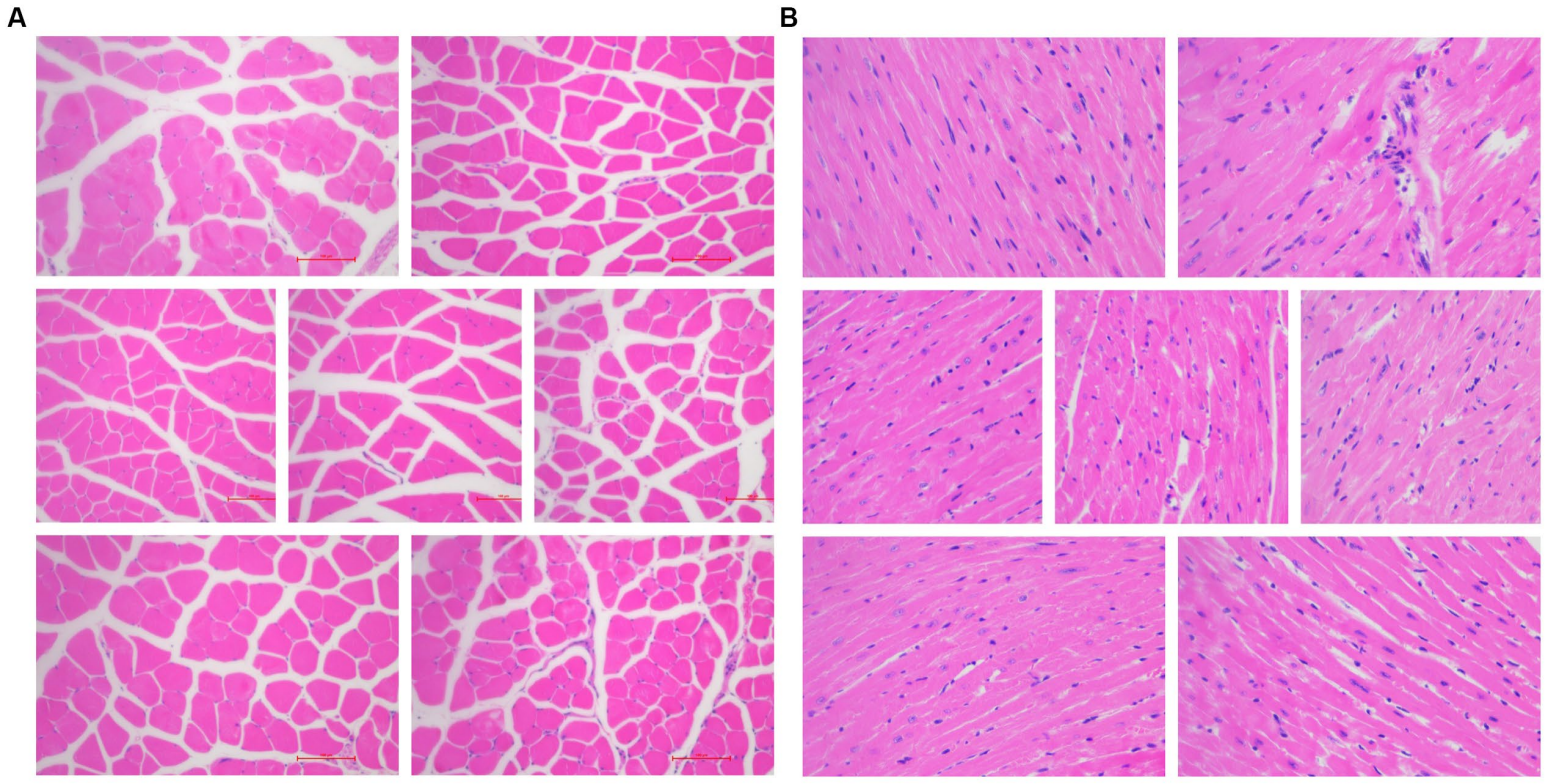
Representative hematoxylin and eosin staining of gastrocnemius muscle and myocardium in different groups. **(A)** Representative HE images of gastrocnemius muscle from the BC, MC, PC, LD, MD, HD, and EX groups. Images were acquired using a 20 × objective; scale bar = 100 μm. **(B)** Representative HE images of myocardium from the BC, MC, PC, LD, MD, HD, and EX groups. Images were acquired using a 40 × objective; scale bar = 100 μm. Representative images were selected from the available HE-stained sections in each group to illustrate the typical morphological features observed for that group. The MC group showed more pronounced histopathological alterations than the BC and EX groups, whereas representative images from cycloastragenol-treated groups appeared to show less severe morphological alterations, with the HD group showing the clearest apparent preservation among the available representative images. Because the available images could not be traced back to individual animals, these observations are presented as qualitative representative findings rather than animal-level semi-quantitative histological data. BC, blank control; MC, model control; PC, positive control; LD, low-dose cycloastragenol; MD, medium-dose cycloastragenol; HD, high-dose cycloastragenol; EX, normoxic exercise.

Representative hematoxylin and eosin staining showed that the gastrocnemius muscle in the BC and EX groups maintained relatively intact architecture and regular fiber arrangement. In contrast, the MC group exhibited more pronounced histopathological alterations, including disorganized muscle fibers, widened inter-fiber spaces, and reduced structural integrity. Compared with the MC group, representative images from the PC, LD, MD, and HD groups appeared to show less severe morphological alterations in gastrocnemius muscle, with the HD group showing the clearest apparent morphological preservation among the available representative images (Figure 7).

Representative myocardial HE staining also revealed clear group differences. The BC and EX groups showed relatively orderly myocardial architecture, whereas the MC group displayed more obvious myocardial fiber disorganization, interstitial widening, and inflammatory cell infiltration. These representative myocardial alterations appeared less severe in the PC, LD, MD, and HD groups, with the HD group showing the clearest apparent preservation among the available representative images (Figure 7). Additional representative HE images are provided in Appendix 2.

### 3.7 Effects of cycloastragenol on HIF-1α/FoxO1 and Nrf2/HO-1-related protein expression

**Figure 8:**
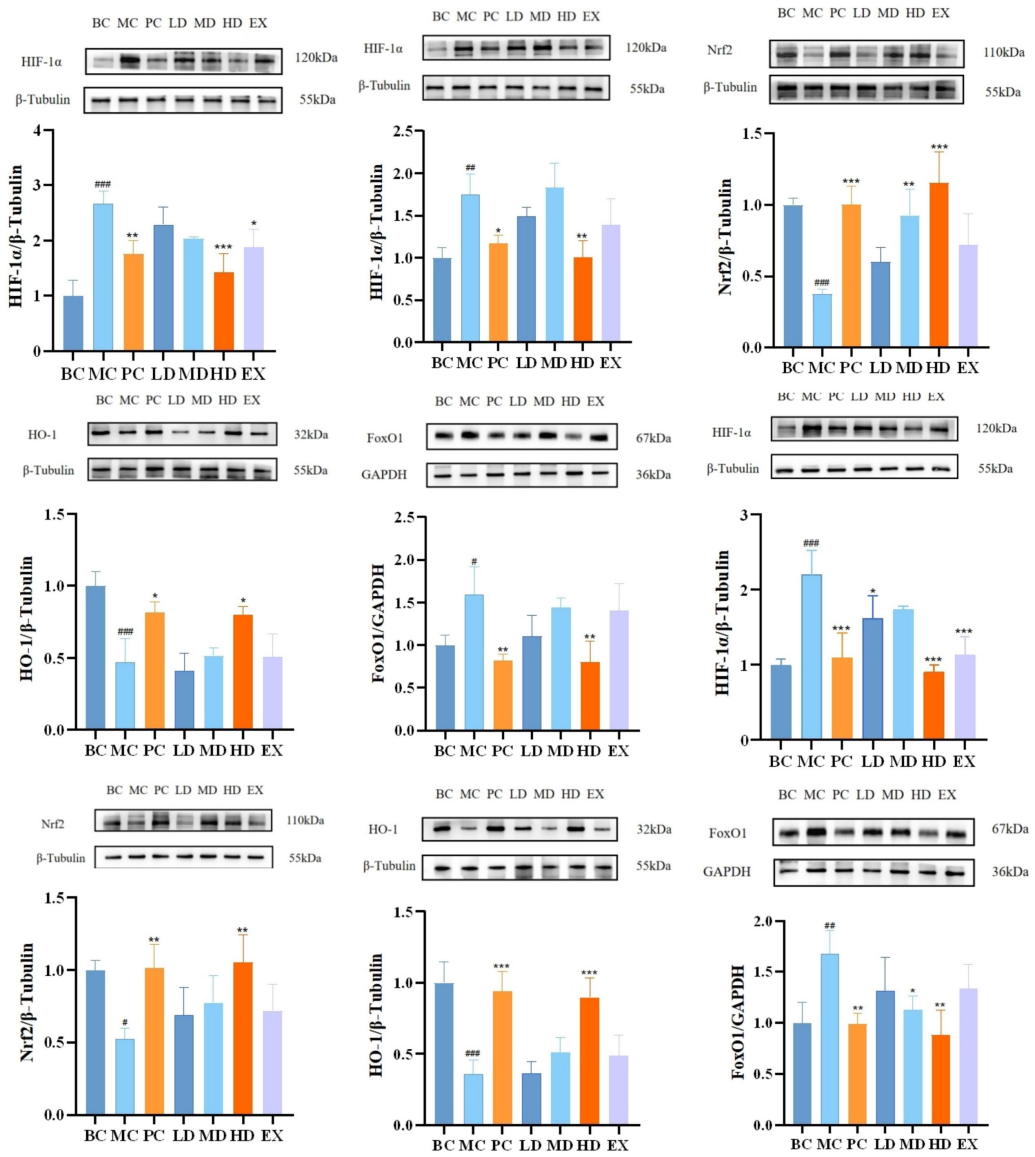
Effects of cycloastragenol on hypoxia-, oxidative stress-, and stress-response-related protein expression. **(A)** HIF-1alpha in brain tissue (*n* = 3 per group). **(B)** HIF-1alpha, Nrf2, HO-1, and FoxO1 in gastrocnemius muscle (*n* = 3 per group). **(C)** HIF-1alpha, Nrf2, HO-1, and FoxO1 in myocardium (*n* = 3 per group). Representative immunoblots and corresponding quantitative analyses are shown. Data are presented as mean ± SD. #, ##, and ### indicate *p* < 0.05, *p* < 0.01, and *p* < 0.001 versus the BC group, respectively; *, **, and *** indicate *p* < 0.05, *p* < 0.01, and *p* < 0.001 versus the MC group, respectively. BC, blank control; MC, model control; PC, positive control; LD, low-dose cycloastragenol; MD, medium-dose cycloastragenol; HD, high-dose cycloastragenol; EX, normoxic exercise.

Western blot analysis showed group-dependent differences in proteins related to hypoxia and oxidative stress responses in brain, gastrocnemius muscle, and myocardium (Figure 8). In brain tissue, normalized HIF-1alpha expression was higher in MC than BC (2.67 ± 0.23 vs. 1.00 ± 0.28). Compared with MC, HIF-1alpha expression was lower in the PC (1.75 ± 0.25; −34.2%; *d* = −3.85; *p* < 0.01) and HD groups (1.42 ± 0.35; −46.6%; *d* = −4.23; *p* < 0.001), indicating attenuation of the hypoxia-related protein response.

In gastrocnemius muscle, MC showed higher HIF-1alpha (1.75 ± 0.24) and FoxO1 (1.59 ± 0.32) and lower Nrf2 (0.38 ± 0.03) and HO-1 (0.47 ± 0.17) expression compared with the BC-normalized reference. Compared with MC, HD cycloastragenol reduced HIF-1alpha (1.01 ± 0.20; −42.6%; *d* = −3.39; *p* < 0.01) and FoxO1 (0.80 ± 0.24; −49.7%; *d* = −2.80; *p* < 0.01), while increasing Nrf2 (1.15 ± 0.22; +206.4%; *d* = +5.02; *p* < 0.001) and HO-1 (0.80 ± 0.06; +70.2%; *d* = +2.69; *p* < 0.05).

A similar but not identical pattern was observed in myocardial tissue. Compared with MC, HD cycloastragenol reduced HIF-1alpha (0.91 ± 0.10 vs. 2.20 ± 0.32; −58.7%; *d* = −5.46; *p* < 0.001) and FoxO1 (0.88 ± 0.24 vs. 1.68 ± 0.23; −47.4%; *d* = −3.43; *p* < 0.01), while increasing Nrf2 (1.05 ± 0.19 vs. 0.52 ± 0.08; +100.8%; *d* = +3.69; *p* < 0.01) and HO-1 (0.90 ± 0.14 vs. 0.36 ± 0.09; +149.4%; *d* = +4.59; *p* < 0.001). These protein expression patterns support the view that cycloastragenol pretreatment was associated with lower hypoxia/FoxO1-related stress signaling and higher antioxidant-response signaling in skeletal muscle and myocardium.

## 4 Discussion

### 4.1 Main findings

The present study provides integrated evidence that cycloastragenol pretreatment was associated with improved tolerance to acute hypoxic exhaustive exercise stress in mice. Overall, cycloastragenol was associated with longer exhaustion time, more favorable metabolic and oxidative stress-related responses, reduced inflammatory markers, and less pronounced representative histopathological alterations in both gastrocnemius muscle and myocardium. In addition, cycloastragenol was associated with changes in HIF-1alpha, FoxO1, Nrf2, and HO-1 expression in brain, skeletal muscle, and myocardial tissues. Importantly, these findings were observed across multiple domains, including exercise performance, circulating biomarkers, tissue morphology, and protein expression, suggesting that the effects of cycloastragenol under acute hypoxic exhaustive exercise conditions were not confined to a single outcome measure but reflected a broader bioactive response to hypoxic exercise stress. However, the protein-expression results should be interpreted as associations rather than proof of pathway mediation.

### 4.2 Exercise performance and metabolic responses under acute hypoxic stress

An important finding of the present study is that cycloastragenol pretreatment was associated with improved exercise performance under acute hypoxic stress, as reflected by the prolonged exhaustion time observed after intervention. This finding is consistent with evidence that acute hypoxic exposure reduces exercise capacity and increases physiological strain (1, 19). It also fits with previous animal studies of related botanical interventions: *Astragalus membranaceus* supplementation improved exercise performance and reduced fatigue-related metabolites in trained mice (20), Astragalus showed anti-fatigue effects in hypoxic mice (21), and Rhodiola crenulata improved exhaustive exercise-induced fatigue in mice (22). Compared with those whole-extract or multi-constituent interventions, the present study narrows the evidence to cycloastragenol, a defined Astragalus-derived constituent, and tests it in a combined 24-h hypobaric hypoxia plus treadmill exhaustion model.

This interpretation is supported by the concurrent changes in BUN and lactate. In previous anti-fatigue studies, improved exercise capacity has often been accompanied by lower lactate, ammonia, or other fatigue-related metabolites after exercise challenge (20, 22). In the present study, BUN and lactate increased after acute hypoxic exhaustive exercise and decreased after cycloastragenol pretreatment, with the strongest effects in the HD group. Because BUN and lactate are commonly used to reflect exercise-related metabolic strain (23), their reduction together with the improvement in exhaustion time suggests that the favorable effect of cycloastragenol on exercise performance was accompanied by a more favorable metabolic response under hypoxic stress. However, because hepatic glycogen, muscle glycogen, and blood glucose were not measured, the present data cannot determine whether cycloastragenol preserved glycogen stores or altered circulating glucose availability. Therefore, the metabolic interpretation should be limited to lactate, BUN, and ATPase-related indices, rather than direct preservation of energy-substrate availability.

### 4.3 Oxidative stress and inflammatory responses under acute hypoxic exhaustive exercise

Another important aspect of the present findings is that cycloastragenol pretreatment was associated with attenuation of oxidative stress induced by acute hypoxic exhaustive exercise (24). In the model group, the decrease in T-SOD activity together with the increase in MDA level indicated impaired antioxidant defense and enhanced lipid peroxidation under hypoxic exercise stress. This pattern is compatible with previous evidence that exhaustive exercise and hypoxic stress can disturb redox homeostasis in skeletal muscle and other tissues, although the magnitude and direction of MDA responses can vary with exercise mode, tissue, and sampling time (25). The reversal of these changes after cycloastragenol pretreatment suggests that cycloastragenol may help preserve redox balance in the setting of acute hypoxic exhaustive exercise.

A similar pattern was observed for inflammatory markers. The elevation of IL-6 and TNF-α after acute hypoxic exhaustive exercise indicates that the stress imposed by the model was not limited to metabolic disturbance but also involved a marked inflammatory response. Cycloastragenol has been reported to show anti-inflammatory and tissue-protective properties in non-exercise injury models, including ischemia-related neuroinflammatory settings (10, 15). The present reductions in IL-6 and TNF-α extend this anti-inflammatory profile to an acute hypoxic exhaustive exercise model. Importantly, the oxidative stress-related and inflammatory changes did not appear as separate findings in the present study; rather, they were moderated in parallel after intervention. This parallel pattern supports the view that cycloastragenol influenced a broader stress-response profile under acute hypoxic exhaustive exercise conditions.

It should also be noted that the normoxic exercise group did not show a statistically significant increase in serum MDA compared with the blank control group in the present experiment. This finding differs from some previous reports showing increased MDA after exhaustive exercise under normoxic conditions. A possible explanation is that MDA responses may vary according to exercise modality, sampling time, biological matrix, tissue specificity, and the degree of exercise stress. In the present study, the oxidative stress response was most evident under the combined hypobaric hypoxia and exhaustive exercise condition. Therefore, the MDA findings should be interpreted as evidence of oxidative stress primarily in the hypoxic exhaustive exercise model rather than as a general response to treadmill exhaustion alone.

### 4.4 Tissue morphology and ATPase-related muscle function

The pattern observed in the present study was not restricted to circulating markers, but also extended to tissue morphology and ATPase-related muscle function. Representative HE staining showed that acute hypoxic exhaustive exercise was accompanied by evident structural alterations in both gastrocnemius muscle and myocardium, whereas representative images from cycloastragenol-treated groups appeared to show less severe morphological abnormalities to different extents. This tissue-level pattern is consistent with previous reports that fatigue- or hypoxia-related stress can involve skeletal muscle and myocardial injury markers, and it supports the interpretation that cycloastragenol effects were not limited to serum-based endpoints.

The ATPase findings further refine this interpretation. In the present dataset, Na + -K + -ATPase activity was clearly reduced by the model and improved by high-dose cycloastragenol, whereas Ca2 + -Mg2 + -ATPase did not show a simple model-induced decrease but was further enhanced in the PC and HD groups. Because Na + -K + -ATPase is closely related to ion transport, excitability, and skeletal muscle metabolic function (26), the improvement in Na + -K + -ATPase activity suggests that cycloastragenol may support muscle ion-transport capacity under hypoxic exercise stress. The Ca2 + -Mg2 + -ATPase result should be interpreted more cautiously as an adaptive or compensatory ATPase-related response rather than simple restoration of a decreased marker. When considered together, the histopathological and ATPase findings suggest that cycloastragenol was associated with less pronounced skeletal muscle structural alteration and more favorable ATPase-related muscle function, while the myocardial HE findings further support a broader tissue-level response.

### 4.5 Network pharmacology and protein expression profiles

The network pharmacology analysis provided a useful framework for interpreting the experimental findings by indicating that the bioactive effects of cycloastragenol may involve multiple targets and pathways related to hypoxic adaptation, inflammatory responses, and stress regulation. Among the enriched pathways, HIF-1, PI3K-Akt, FoxO, and IL-17 signaling were highlighted as potentially relevant. These pathways overlap with known biological themes in hypoxia/exercise stress and with previous work on Astragalus-derived compounds, which have been linked to antioxidant, anti-inflammatory, and tissue-protective responses (8, 15). These analyses should be regarded as predictive rather than confirmatory; however, they were useful for identifying biologically plausible pathways that could be compared with the subsequent protein expression results.

The Western blot findings further supported the relevance of hypoxia- and oxidative stress-related signaling in the present model. In brain, skeletal muscle, and myocardial tissues, cycloastragenol pretreatment was associated with reduced HIF-1alpha expression, while in skeletal muscle and myocardium it was also associated with increased Nrf2 and HO-1 expression and reduced FoxO1 expression. These changes are directionally consistent with previous evidence that exercise and oxidative stress can engage Nrf2-related antioxidant responses (25), and with reports that cycloastragenol can attenuate oxidative and inflammatory injury responses in other experimental contexts (10, 15). At the same time, the current findings do not validate all pathways predicted by network pharmacology. In particular, PI3K-Akt and IL-17 signaling were not directly assessed in the present study, and their roles therefore remain speculative. Accordingly, the combined findings are best interpreted as suggesting that cycloastragenol may be associated, at least in part, with coordinated modulation of hypoxia-related and oxidative stress-responsive pathways, while additional mechanistic studies are still needed.

### 4.6 Practical implications and study limitations

The present findings indicate that cycloastragenol may warrant further investigation as a bioactive nutritional candidate in experimental settings involving acute hypoxic exercise stress. In this study, its effects were reflected across several interconnected domains, including exercise performance, metabolic and inflammatory responses, tissue morphology, and protein expression. This multi-level pattern is noteworthy because it suggests that the response associated with cycloastragenol was not restricted to a single biochemical or functional index, but extended more broadly to the overall physiological response to acute hypoxic exhaustive exercise. However, these findings should be interpreted within the limits of an experimental animal study and should not be directly extrapolated to humans or practical high-altitude applications without further evidence.

Several limitations of the present study should be acknowledged. First, only male mice were included, which limits the generalizability of the findings and does not address potential sex differences in hypoxic adaptation, oxidative stress responses, inflammatory regulation, or exercise performance. Second, the model represented acute rather than chronic hypoxic exposure and therefore may not fully reflect longer-term adaptation to hypoxic environments. Third, hepatic glycogen, muscle glycogen, and blood glucose were not measured. These endpoints are important for directly evaluating exercise-related energy reserves, and their absence limits the strength of conclusions regarding the substrate basis of the observed improvement in exercise tolerance. Fourth, the histopathological findings were based on representative images and were not subjected to formal animal-level semi-quantitative statistical analysis because the available images could not be matched to individual animals. Fifth, although sample testing was performed under single-blind conditions, the treadmill exhaustion endpoint was not fully blinded and the exact shock-grid current was not recorded in the original experimental log. Sixth, ELISA and Western blot analyses had limited effective sample sizes, and some Western blot procedural records, such as protein loading amount, membrane type, antibody dilutions, exposure time, and independent blot count, could not be fully reconstructed from the available files. Seventh, tissue samples were collected immediately at exhaustion; because exhaustion time differed among groups, protein expression endpoints such as HIF-1alpha, Nrf2, HO-1, and FoxO1 may partly reflect differences in exercise exposure time and physiological stress at sampling. Eighth, the network pharmacology analysis was based on human gene symbols and *Homo sapiens* STRING identifiers; a formal human-to-mouse orthologue conversion workflow was not documented, so animal experiments should be interpreted as downstream biological validation of selected pathway-associated proteins rather than direct validation of all predicted targets. Finally, although network pharmacology and Western blot results provided useful mechanistic clues, no inhibitor-based, gene-intervention, phosphorylation, or target-engagement experiments were performed to establish causal relationships between specific signaling pathways and the observed responses. Accordingly, further studies are needed to clarify the molecular and metabolic basis of cycloastragenol action and to determine whether these findings can be translated to broader physiological or applied settings.

## 5 Conclusion

Cycloastragenol pretreatment was associated with improved tolerance to acute hypoxic exhaustive exercise stress in mice. This response was reflected in longer exhaustion time, more favorable lactate, BUN, oxidative stress, and inflammatory profiles, and less pronounced representative histopathological alterations in gastrocnemius muscle and myocardium. In addition, cycloastragenol was associated with modulation of HIF-1α, FoxO1, Nrf2, and HO-1 expression in brain, skeletal muscle, and myocardial tissues. Taken together, the present findings suggest that cycloastragenol may have potential as a bioactive nutritional candidate for acute hypoxic exercise stress. However, given the limitations of the present animal model, the absence of direct hepatic glycogen, muscle glycogen, and blood glucose measurements, and the lack of causal mechanistic verification, further studies are needed to clarify the underlying molecular and metabolic basis and evaluate the broader applicability of these findings.

## Data Availability

The raw data supporting the conclusions of this article will be made available by the authors, without undue reservation.
