# Supplementary material for: Cycloastragenol improves tolerance to acute hypoxic exhaustive exercise stress in mice: integrated network pharmacology and experimental evidence
Source: Front Nutr. 2026 Jul 23;13:1856961. doi: 10.3389/fnut.2026.1856961 (PMC13446268; doi:10.3389/fnut.2026.1856961)
Supplement: Supplementary file 1 [file Supplementary_file_2.docx]

Supplementary Figures and Analysis Images

Main_Manuscript_Figures_Album

# Main Manuscript Figures Album

Image count: 10

Each caption records the original source filename/path from the revision package.

Analysis_Figure_Exports_Album

# Analysis Figure Export Images Album

Image count: 23

Each caption records the original source filename/path from the revision package.

**03_Supplementary_Files/Figures/Analysis_Figure_Exports/Color/BUN.emf**

**03_Supplementary_Files/Figures/Analysis_Figure_Exports/Color/CaMg_ATPase.emf**

**03_Supplementary_Files/Figures/Analysis_Figure_Exports/Color/Exhaustion_time.emf**

**03_Supplementary_Files/Figures/Analysis_Figure_Exports/Color/IL6.emf**

**03_Supplementary_Files/Figures/Analysis_Figure_Exports/Color/LAC.emf**

**03_Supplementary_Files/Figures/Analysis_Figure_Exports/Color/MDA.emf**

**03_Supplementary_Files/Figures/Analysis_Figure_Exports/Color/MDA_2.emf**

**03_Supplementary_Files/Figures/Analysis_Figure_Exports/Color/NaK_ATPase.emf**

**03_Supplementary_Files/Figures/Analysis_Figure_Exports/Color/T_SOD.emf**

**03_Supplementary_Files/Figures/Analysis_Figure_Exports/Color/T_SOD_2.emf**

**03_Supplementary_Files/Figures/Analysis_Figure_Exports/Color/TNFalpha.emf**

**03_Supplementary_Files/Figures/Analysis_Figure_Exports/Grayscale/BUN.emf**

**03_Supplementary_Files/Figures/Analysis_Figure_Exports/Grayscale/CaMg_ATPase.emf**

**03_Supplementary_Files/Figures/Analysis_Figure_Exports/Grayscale/Exhaustion_time.emf**

**03_Supplementary_Files/Figures/Analysis_Figure_Exports/Grayscale/IL6.emf**

**03_Supplementary_Files/Figures/Analysis_Figure_Exports/Grayscale/LAC.emf**

**03_Supplementary_Files/Figures/Analysis_Figure_Exports/Grayscale/MDA.emf**

**03_Supplementary_Files/Figures/Analysis_Figure_Exports/Grayscale/MDA_2.emf**

**03_Supplementary_Files/Figures/Analysis_Figure_Exports/Grayscale/NaK_ATPase.emf**

**03_Supplementary_Files/Figures/Analysis_Figure_Exports/Grayscale/T_SOD.emf**

**03_Supplementary_Files/Figures/Analysis_Figure_Exports/Grayscale/T_SOD_2.emf**

**03_Supplementary_Files/Figures/Analysis_Figure_Exports/Grayscale/TNFalpha.emf**

**03_Supplementary_Files/Figures/Analysis_Figure_Exports/Other/Body_weight.emf**

Network_Pharmacology_Figures_Album

# Network Pharmacology Figures Album

Image count: 22

Each caption records the original source filename/path from the revision package.

**03_Supplementary_Files/Network_Pharmacology/Hypoxia_Cycloastragenol_Target_Screening/Enrichment_Figures/GO_BP.png**

**03_Supplementary_Files/Network_Pharmacology/Hypoxia_Cycloastragenol_Target_Screening/Enrichment_Figures/GO_CC.png**

**03_Supplementary_Files/Network_Pharmacology/Hypoxia_Cycloastragenol_Target_Screening/Enrichment_Figures/GO_MF.png**

**03_Supplementary_Files/Network_Pharmacology/Hypoxia_Cycloastragenol_Target_Screening/Enrichment_Figures/KEGG.png**

**03_Supplementary_Files/Network_Pharmacology/Hypoxia_Cycloastragenol_Target_Screening/STRING_PPI/PPI_network_1.png**

**03_Supplementary_Files/Network_Pharmacology/Hypoxia_Cycloastragenol_Target_Screening/STRING_PPI/PPI_network_2.png**

**03_Supplementary_Files/Network_Pharmacology/Hypoxia_Cycloastragenol_Target_Screening/STRING_PPI/PPI_network_3.png**

**03_Supplementary_Files/Network_Pharmacology/Hypoxia_Cycloastragenol_Target_Screening/STRING_PPI/PPI_network_score1.png**

**03_Supplementary_Files/Network_Pharmacology/Hypoxia_Cycloastragenol_Target_Screening/STRING_PPI/PPI_network_score2.png**

**03_Supplementary_Files/Network_Pharmacology/Hypoxia_Cycloastragenol_Target_Screening/STRING_PPI/PPI_network_score3.png**

**03_Supplementary_Files/Network_Pharmacology/Hypoxia_Cycloastragenol_Target_Screening/STRING_PPI/STRING_high_resolution_network.png**

**03_Supplementary_Files/Network_Pharmacology/Hypoxia_Cycloastragenol_Target_Screening/STRING_PPI/STRING_interactions_preview.png**

**03_Supplementary_Files/Network_Pharmacology/Network_Pharmacology_Supplement/DAVID_Enrichment/DAVID_116_targets_GO_BP_bubble_plot.png**

**03_Supplementary_Files/Network_Pharmacology/Network_Pharmacology_Supplement/DAVID_Enrichment/DAVID_116_targets_GO_CC_bubble_plot.png**

**03_Supplementary_Files/Network_Pharmacology/Network_Pharmacology_Supplement/DAVID_Enrichment/DAVID_116_targets_GO_MF_bubble_plot.png**

**03_Supplementary_Files/Network_Pharmacology/Network_Pharmacology_Supplement/DAVID_Enrichment/DAVID_116_targets_KEGG_bubble_plot.png**

**03_Supplementary_Files/Network_Pharmacology/Network_Pharmacology_Supplement/DAVID_Enrichment/DAVID_29_targets_GO_BP_bubble_plot.png**

**03_Supplementary_Files/Network_Pharmacology/Network_Pharmacology_Supplement/DAVID_Enrichment/DAVID_29_targets_GO_CC_bubble_plot.png**

**03_Supplementary_Files/Network_Pharmacology/Network_Pharmacology_Supplement/DAVID_Enrichment/DAVID_29_targets_GO_MF_bubble_plot.png**

**03_Supplementary_Files/Network_Pharmacology/Network_Pharmacology_Supplement/DAVID_Enrichment/DAVID_29_targets_KEGG_bubble_plot.png**

**03_Supplementary_Files/Network_Pharmacology/Network_Pharmacology_Supplement/DAVID_Enrichment/DAVID_STRING_0_9_KEGG_bubble_plot.png**

**03_Supplementary_Files/Network_Pharmacology/Network_Pharmacology_Supplement/Overlap_Analysis/jVenn_chart.png**

Western_Blot_Figure_Exports_Album

# Western Blot Figure Export Images Album

Image count: 18

Each caption records the original source filename/path from the revision package.

**03_Supplementary_Files/Western_Blot/Figure_Exports/FOXO.emf**

**03_Supplementary_Files/Western_Blot/Figure_Exports/FOXO1.emf**

**03_Supplementary_Files/Western_Blot/Figure_Exports/FOXO1_2.emf**

**03_Supplementary_Files/Western_Blot/Figure_Exports/FOXO_2.emf**

**03_Supplementary_Files/Western_Blot/Figure_Exports/HIF-1.emf**

**03_Supplementary_Files/Western_Blot/Figure_Exports/HIF-1_2.emf**

**03_Supplementary_Files/Western_Blot/Figure_Exports/HIF-1_3.emf**

**03_Supplementary_Files/Western_Blot/Figure_Exports/HIF-1_4.emf**

**03_Supplementary_Files/Western_Blot/Figure_Exports/HIF-1_5.emf**

**03_Supplementary_Files/Western_Blot/Figure_Exports/HIF-1_6.emf**

**03_Supplementary_Files/Western_Blot/Figure_Exports/HO-1.emf**

**03_Supplementary_Files/Western_Blot/Figure_Exports/HO-1_2.emf**

**03_Supplementary_Files/Western_Blot/Figure_Exports/HO-1_3.emf**

**03_Supplementary_Files/Western_Blot/Figure_Exports/HO-1_4.emf**

**03_Supplementary_Files/Western_Blot/Figure_Exports/Nrf2.emf**

**03_Supplementary_Files/Western_Blot/Figure_Exports/Nrf2_2.emf**

**03_Supplementary_Files/Western_Blot/Figure_Exports/Nrf2_3.emf**

**03_Supplementary_Files/Western_Blot/Figure_Exports/Nrf2_4.emf**
