# Supplementary material for: Cycloastragenol improves tolerance to acute hypoxic exhaustive exercise stress in mice: integrated network pharmacology and experimental evidence
Source: Front Nutr. 2026 Jul 23;13:1856961. doi: 10.3389/fnut.2026.1856961 (PMC13446268; doi:10.3389/fnut.2026.1856961)
Supplement: Supplementary file 2 [file Supplementary_file_3.docx]

Supplementary Raw HE and Western Blot Images

# Figure 7A Raw Gastrocnemius HE Images Album

Image count: 7

Each caption records the original source filename/path from the revision package.

# Figure 7B Raw Myocardium HE Images Album

Image count: 7

Each caption records the original source filename/path from the revision package.

Western_Blot_Raw_Images_Album

# Western Blot Raw Images Album

Image count: 54

Each caption records the original source filename/path from the revision package.

**03_Supplementary_Files/Western_Blot/Raw_Blot_Images/brain_beta_tubulin_loading_control_for_HIF1alpha_rep1.tif**

**03_Supplementary_Files/Western_Blot/Raw_Blot_Images/brain_beta_tubulin_loading_control_for_HIF1alpha_rep3.tif**

**03_Supplementary_Files/Western_Blot/Raw_Blot_Images/brain_HIF1alpha_rep1.tif**

**03_Supplementary_Files/Western_Blot/Raw_Blot_Images/brain_HIF1alpha_rep2.tif**

**03_Supplementary_Files/Western_Blot/Raw_Blot_Images/brain_HIF1alpha_rep2_2.tif**

**03_Supplementary_Files/Western_Blot/Raw_Blot_Images/brain_HIF1alpha_rep3.tif**

**03_Supplementary_Files/Western_Blot/Raw_Blot_Images/gastrocnemius_beta_tubulin_loading_control_for_HIF1alpha_rep1.tif**

**03_Supplementary_Files/Western_Blot/Raw_Blot_Images/gastrocnemius_beta_tubulin_loading_control_for_HIF1alpha_rep2.tif**

**03_Supplementary_Files/Western_Blot/Raw_Blot_Images/gastrocnemius_beta_tubulin_loading_control_for_HIF1alpha_rep3.tif**

**03_Supplementary_Files/Western_Blot/Raw_Blot_Images/gastrocnemius_beta_tubulin_loading_control_for_HO1_rep1.tif**

**03_Supplementary_Files/Western_Blot/Raw_Blot_Images/gastrocnemius_beta_tubulin_loading_control_for_HO1_rep2.tif**

**03_Supplementary_Files/Western_Blot/Raw_Blot_Images/gastrocnemius_beta_tubulin_loading_control_for_HO1_rep3.tif**

**03_Supplementary_Files/Western_Blot/Raw_Blot_Images/gastrocnemius_beta_tubulin_loading_control_for_Nrf2_rep1.tif**

**03_Supplementary_Files/Western_Blot/Raw_Blot_Images/gastrocnemius_beta_tubulin_loading_control_for_Nrf2_rep2.tif**

**03_Supplementary_Files/Western_Blot/Raw_Blot_Images/gastrocnemius_beta_tubulin_loading_control_for_Nrf2_rep3.tif**

**03_Supplementary_Files/Western_Blot/Raw_Blot_Images/gastrocnemius_FOXO1_rep1.tif**

**03_Supplementary_Files/Western_Blot/Raw_Blot_Images/gastrocnemius_FOXO1_rep2.tif**

**03_Supplementary_Files/Western_Blot/Raw_Blot_Images/gastrocnemius_FOXO1_rep3.tif**

**03_Supplementary_Files/Western_Blot/Raw_Blot_Images/gastrocnemius_GAPDH_loading_control_for_FOXO1_rep1.tif**

**03_Supplementary_Files/Western_Blot/Raw_Blot_Images/gastrocnemius_GAPDH_loading_control_for_FOXO1_rep2.tif**

**03_Supplementary_Files/Western_Blot/Raw_Blot_Images/gastrocnemius_GAPDH_loading_control_for_FOXO1_rep3.tif**

**03_Supplementary_Files/Western_Blot/Raw_Blot_Images/gastrocnemius_HIF1alpha_rep1.tif**

**03_Supplementary_Files/Western_Blot/Raw_Blot_Images/gastrocnemius_HIF1alpha_rep2.tif**

**03_Supplementary_Files/Western_Blot/Raw_Blot_Images/gastrocnemius_HIF1alpha_rep3.tif**

**03_Supplementary_Files/Western_Blot/Raw_Blot_Images/gastrocnemius_HO1_rep1.tif**

**03_Supplementary_Files/Western_Blot/Raw_Blot_Images/gastrocnemius_HO1_rep2.tif**

**03_Supplementary_Files/Western_Blot/Raw_Blot_Images/gastrocnemius_HO1_rep3.tif**

**03_Supplementary_Files/Western_Blot/Raw_Blot_Images/gastrocnemius_Nrf2_rep1.tif**

**03_Supplementary_Files/Western_Blot/Raw_Blot_Images/gastrocnemius_Nrf2_rep2.tif**

**03_Supplementary_Files/Western_Blot/Raw_Blot_Images/gastrocnemius_Nrf2_rep3.tif**

**03_Supplementary_Files/Western_Blot/Raw_Blot_Images/myocardium_beta_tubulin_loading_control_for_HIF1alpha_rep1.tif**

**03_Supplementary_Files/Western_Blot/Raw_Blot_Images/myocardium_beta_tubulin_loading_control_for_HIF1alpha_rep2.tif**

**03_Supplementary_Files/Western_Blot/Raw_Blot_Images/myocardium_beta_tubulin_loading_control_for_HIF1alpha_rep3.tif**

**03_Supplementary_Files/Western_Blot/Raw_Blot_Images/myocardium_beta_tubulin_loading_control_for_HO1_rep1.tif**

**03_Supplementary_Files/Western_Blot/Raw_Blot_Images/myocardium_beta_tubulin_loading_control_for_HO1_rep2.tif**

**03_Supplementary_Files/Western_Blot/Raw_Blot_Images/myocardium_beta_tubulin_loading_control_for_HO1_rep3.tif**

**03_Supplementary_Files/Western_Blot/Raw_Blot_Images/myocardium_beta_tubulin_loading_control_for_Nrf2_rep1.tif**

**03_Supplementary_Files/Western_Blot/Raw_Blot_Images/myocardium_beta_tubulin_loading_control_for_Nrf2_rep2.tif**

**03_Supplementary_Files/Western_Blot/Raw_Blot_Images/myocardium_beta_tubulin_loading_control_for_Nrf2_rep3.tif**

**03_Supplementary_Files/Western_Blot/Raw_Blot_Images/myocardium_FOXO1_rep1.tif**

**03_Supplementary_Files/Western_Blot/Raw_Blot_Images/myocardium_FOXO1_rep2.tif**

**03_Supplementary_Files/Western_Blot/Raw_Blot_Images/myocardium_FOXO1_rep3.tif**

**03_Supplementary_Files/Western_Blot/Raw_Blot_Images/myocardium_GAPDH_loading_control_for_FOXO1_rep1.tif**

**03_Supplementary_Files/Western_Blot/Raw_Blot_Images/myocardium_GAPDH_loading_control_for_FOXO1_rep2.tif**

**03_Supplementary_Files/Western_Blot/Raw_Blot_Images/myocardium_GAPDH_loading_control_for_FOXO1_rep3.tif**

**03_Supplementary_Files/Western_Blot/Raw_Blot_Images/myocardium_HIF1alpha_rep1.tif**

**03_Supplementary_Files/Western_Blot/Raw_Blot_Images/myocardium_HIF1alpha_rep2.tif**

**03_Supplementary_Files/Western_Blot/Raw_Blot_Images/myocardium_HIF1alpha_rep3.tif**

**03_Supplementary_Files/Western_Blot/Raw_Blot_Images/myocardium_HO1_rep1.tif**

**03_Supplementary_Files/Western_Blot/Raw_Blot_Images/myocardium_HO1_rep2.tif**

**03_Supplementary_Files/Western_Blot/Raw_Blot_Images/myocardium_HO1_rep3.tif**

**03_Supplementary_Files/Western_Blot/Raw_Blot_Images/myocardium_Nrf2_rep1.tif**

**03_Supplementary_Files/Western_Blot/Raw_Blot_Images/myocardium_Nrf2_rep2.tif**

**03_Supplementary_Files/Western_Blot/Raw_Blot_Images/myocardium_Nrf2_rep3.tif**
